# Genomic epidemiology of the first epidemic wave of severe acute respiratory syndrome coronavirus 2 (SARS-CoV-2) in Palestine

**DOI:** 10.1099/mgen.0.000584

**Published:** 2021-06-22

**Authors:** Nouar Qutob, Zaidoun Salah, Damien Richard, Hisham Darwish, Husam Sallam, Issa Shtayeh, Osama Najjar, Mahmoud Ruzayqat, Dana Najjar, François Balloux, Lucy van Dorp

**Affiliations:** ^1^​ Department of Health Sciences, Faculty of Graduate Studies, Arab American University, Ramallah, Palestine; ^2^​ Institute of Child Health, University College London, London, UK; ^3^​ UCL Genetics Institute, University College London, London, UK; ^4^​ Palestinian Ministry of Health, Ramallah, Palestine; ^§^​ Present address: Al Quds Bard College, Al-Quds University, East Jerusalem, Palestine

**Keywords:** COVID-19, genomic epidemiology, minor allele frequency, phylogenetics, within-host genetic diversity

## Abstract

Severe acute respiratory syndrome coronavirus 2 (SARS-CoV-2), the novel coronavirus responsible for the COVID-19 pandemic, continues to cause a significant public-health burden and disruption globally. Genomic epidemiology approaches point to most countries in the world having experienced many independent introductions of SARS-CoV-2 during the early stages of the pandemic. However, this situation may change with local lockdown policies and restrictions on travel, leading to the emergence of more geographically structured viral populations and lineages transmitting locally. Here, we report the first SARS-CoV-2 genomes from Palestine sampled from early March 2020, when the first cases were observed, through to August of 2020. SARS-CoV-2 genomes from Palestine fall across the diversity of the global phylogeny, consistent with at least nine independent introductions into the region. We identify one locally predominant lineage in circulation represented by 50 Palestinian SARS-CoV-2, grouping with genomes generated from Israel and the UK. We estimate the age of introduction of this lineage to 05/02/2020 (16/01/2020–19/02/2020), suggesting SARS-CoV-2 was already in circulation in Palestine predating its first detection in Bethlehem in early March. Our work highlights the value of ongoing genomic surveillance and monitoring to reconstruct the epidemiology of COVID-19 at both local and global scales.

## Data Summary

All newly generated assemblies have been uploaded to GISAID (https://www.epicov.org) and are available upon registration under IDs EPI_ISL_596500–EPI_ISL_596568. In addition, raw short reads have been uploaded to the National Center for Biotechnology Information Sequence Read Archive (SRA) under BioProject accession number PRJNA669945. Information on all samples, including the global accession numbers used in the analysis, are provided in Table S1 (available in the online version of this article).

**Impact Statement**

Genomic epidemiology is a valuable tool to reconstruct the spread of severe acute respiratory syndrome coronavirus 2 (SARS-CoV-2) in different settings. In this work, we generate genomic data for 69 SARS-CoV-2 samples from patients in Palestine, a region until now underrepresented by genomic-surveillance initiatives. Samples spanned from early March through to August 2020, allowing us to provide characterization of the genomic diversity of SARS-CoV-2 in Palestine over the first 2020 epidemic wave of COVID-19. Considering our data in the context of a global dataset of over 50 000 SARS-CoV-2 genomes sampled up until August 2020, we could identify at least nine independent introductions of SARS-CoV-2 into Palestine. Among these, we could phylogenetically resolve a local transmission cluster including 50 SARS-CoV-2 samples from the region. We estimate this local transmission cluster dates to early February 2020, preceding the first confirmed COVID-19 cases in Palestine in early March 2020. Our findings highlight the value of genomic epidemiology approaches to understand the constantly changing transmission dynamics of SARS-CoV-2, at both local and global scales.

## Introduction

Severe acute respiratory syndrome coronavirus 2 (SARS-CoV-2), the novel coronavirus responsible for the coronavirus disease 2019 (COVID-19) pandemic, has spread rapidly around the world since its emergence towards the end of 2019 in China [1–3]. Thanks to the massive efforts of public-health agencies and research teams throughout the world, a very large number of genome assemblies have been made available and allowed the following of the dynamic of the pandemic, essentially in real time [4, 5]. This large and growing resource has brought genomics to the forefront as a method to understand both the ongoing evolution of the virus, but also as a surveillance and epidemiological tool [6].

Genomic data can be a rich source of information to inform on a variety of key epidemiological parameters, such as the age and geographical origins of epidemics, their relative growth rates, to distinguish persistent infections from reinfections, and to inform on the relative contributions of imported cases compared to sustained community or cryptic transmission. A wealth of genomic studies of SARS-CoV-2 from the more local [3, 7–16] through to continental [17, 18] and global scales [1, 19] have consistently pointed to most densely sequenced countries around the world having experienced a number of independent introductions, seeding local transmission chains that are subsequently maintained or may go extinct. For example, analyses of genomic data from the UK’s early 2020 epidemic wave identified over 1000 imported transmission lineages of SARS-CoV-2, with lineage diversity in the UK peaking in late March 2020 [10]. Analyses of the early Washington State (USA) outbreak could identify, using a spatial Bayesian framework, introductions from Hubei province, China, in late January to early February 2020; with similarly early outbreaks in Northern Italy likely deriving from introductions from China over a comparable time period [17].

With the use of non-pharmaceutical interventions to tackle COVID-19, including travel bans, social distancing measures and local/nationwide lockdowns, the patterns of SARS-CoV-2 transmission may be altered from that reconstructed very early in the pandemic. In particular, genomic epidemiology studies of viruses sampled in mid to late 2020 identified the presence of more closely related sets of viruses in circulation, which may define within-country spatial infection clusters, sometimes deriving from known close-contact events [20]. For instance, the reappearance of SARS-CoV-2 in New Zealand in October 2020 despite the virus not having been observed for 102 days prior to its re-emergence in the community. While SARS-CoV-2 samples collected in New Zealand during the ‘first wave’ derived from multiple imports, predominately from North America [16], early analysis of samples collected during the August 2020 outbreak suggest the secondary outbreak consists largely of closely related viruses assigned to the B.1.1.1 lineage (https://nextstrain.org/ncov/oceania?c=region) [6].

Some regions of the globe have conducted extensive genomic surveillance of SARS-CoV-2. For example, the UK viral population has been sampled to unprecedented depth (>238 000 complete assemblies on GISAID as of 17/02/2021). Conversely, the diversity of SARS-CoV-2 circulating in other regions of the world remains under sampled and under studied. A wider geographical coverage of SARS-CoV-2, including genomic samples from additional countries, is valuable as it may in time facilitate comparisons over many nations, characterized by different climate, pandemic mitigation strategies and human population densities, as well as the age/health status of the general population. It is also vital for the early identification of emerging lineages of concern.

**Fig. 1.**
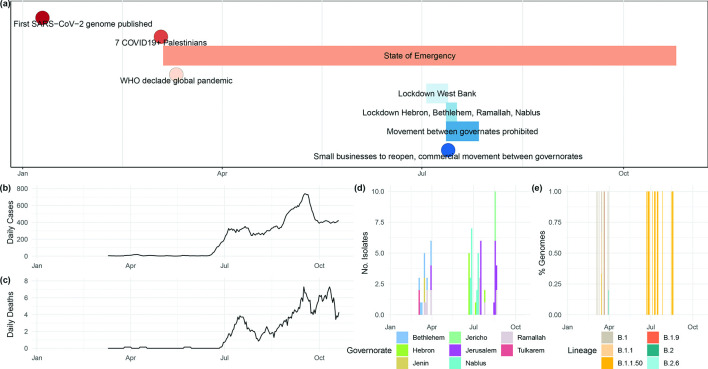
(a) Timeline of interventions in Palestine (as referenced in the text) over the first epidemic wave of SARS-CoV-2 in the region. Plotted using the R package vistime (https://cran.r-project.org/web/packages/vistime/index.html). (**b**) The daily case counts and (c) daily death counts over the time span discussed in the main text, obtained from ourworldindata.org [45]. (**d**) Number of SARS-CoV-2 genomes generated by sampling date (*x*-axis) coloured by the governorate and (e) pango lineage (%) obtained for each sample. A key with the colour schemes for (d) and (e) is provided at the bottom of the figure. Full metadata for all novel data presented in this study are provided in Table S1.

The government of Palestine declared an emergency period for 1 month on March 5th 2020, after seven Palestinians tested positive for SARS-CoV-2 in Bethlehem on March 4th 2020 (Fig. 1). A curfew was declared, quarantining the population except in cases of emergency. The state of emergency was extended for a further month. On May 25th 2020, the restrictions were eased following a decline in cases and a reduction of the rate of positive tests in Palestinian workers returning from Israeli areas. Seroprevalence, as measured up to July 2020, remained low [21].

However, cases surged again during July 2020, with the epicentre of the epidemic in Hebron accounting for over 70% of active cases. On the 3rd of July 2020, a 10 day complete lockdown was declared across the entire West Bank. On the 12th of July 2020, a complete lockdown for 5 days was declared in Hebron, Bethlehem, Ramallah and Nablus governorates. Movement between all governorates was prohibited until the 27th July 2020, with a night-time and weekend curfew imposed on residents except for a few permitted services. All social public gatherings and transportation between governorates were prohibited. However, after the 13th of July 2020, the government of Palestine announced an ease in the restrictions allowing small businesses to reopen, subject to restrictions, and commercial movement between governorates (Fig. 1a). An existing state of emergency was extended since March 2020 with partial lockdowns and school closures implemented during the 20th of December 2020 and the 17th of January 2021 [22]. By the 1st of March 2021, 210 073 cases and 2275 deaths had been reported by the Palestinian Ministry of Health [23].

To better understand the epidemiology of early introductions and transmission of SARS-CoV-2 in Palestine, through to the spring epidemic and its aftermath until late summer, we generated high-quality genomic assemblies for 69 SARS-CoV-2 sampled from patients between the 4th of March 2020 and the 19th of August 2020 (Fig. 1d, e). We phylogenetically placed these samples in the context of 54 804 global SARS-CoV-2 genomes available on GISAID [4, 5] at the end of August 2020 (25/08/2020). This allowed us to quantify the minimum number of introductions of SARS-CoV-2 into Palestine and to identify a sizeable local transmission cluster, sustained since its appearance, which we estimate to significantly predate the first documented COVID-19 cases in Palestine.

## Methods

### Data collection and processing

Nasopharyngeal swabs were sampled between the 4th of March 2020 and the 19th of August 2020 from a sample of 300 Palestinian COVID-19 patients originating from 17 locations within eight governorates (Fig. 1d, e, Table S1). A governate defines a Palestinian administrative district, which may comprise more than one geographical location. RNA was extracted from clinical samples using a QIAamp MinElute virus spin kit. Real-time reverse transcriptase (RT)-PCR was used to detect SARS-CoV-2 using the Seegene company Allplex 2019-nCoV assay. All specimens were handled under a biosafety cabinet according to laboratory biosafety guidelines. For four samples (60, 61, 62, 96), for which only information on the month of sample collection was available, the collection date was set to the middle (15th) of the month. Information on the timing of interventions in the region was obtained from consultation with co-authors and Palestinian Ministry of Health records (http://site.moh.ps).

### Palestine SARS-CoV-2 dataset: sequencing and variant calling

SARS-CoV-2 samples from 96 Palestinian COVID-19 patients with *C*_t_ values ranging between 9 and 30 were chosen strategically to cover a time span between 4th of March 2020 and the 19th of August 2020 and locations within the governorates of Palestine where cases were detected during that period. cDNA synthesis was done using the NEBNext non-directional RNA-Seq workflow and NEBNext Ultra RNA first strand synthesis module and the NEBNext RNA second strand synthesis module. Library preparation was performed with the Nextera Flex for Enrichment workflow [24]. Sequencing was performed on an Illumina NextSeq 550 sequencing apparatus. The mapping of the raw sequencing reads to the Wuhan-Hu-1 reference sequence (GenBank accession no. MN908947; equivalent GISAID ID EPI_ISL_402125) was performed using the Dragen RNA pathogen detection v.3.5.14 pipeline [25]. Strains displayed a mean coverage ranging from 22× to 9400× (Table S1, Fig. S1), with 69 samples considered of sufficient coverage for downstream analysis. Variants were called using Freebayes v1.22 [26]. Three distinct indels (each present in less than three sequences) and 762 unfiltered variable sites were identified. We initially called all SNPs regardless of intra-genome frequency, taking forwards those supported by an intra-genome frequency of at least 0.65 for phylogenetic analysis, but using a lower threshold of 0.05 to create a SNP set dedicated to the study of minor allele frequencies. We carefully inspected that all minor alleles called were supported by multiple sequencing reads from multiple read pairs and after removal of PCR duplicates. SNPs flagged as putative sequencing artefacts were masked (a full list of masked sites is available at https://github.com/W-L/ProblematicSites_SARS-CoV2/blob/master/problematic_sites_sarsCov2.vcf, accessed 25/08/2020) [27, 28]. Across the final dataset, we obtained 128 high-quality SNPs (Fig. S2, Table S2).

### Worldwide SARS-CoV-2 dataset

Additionally, we downloaded 56 803 high-quality assemblies (high coverage, >29 700 bp and with a fraction of ‘N’ nucleotides <5%) from the worldwide SARS-CoV-2 diversity available on GISAID [4, 5] on 25/08/2020, to span the first epidemic wave. All animal-associated genomes were removed, as well as samples flagged by NextStrain as ‘exclude’ (https://github.com/nextstrain/ncov/blob/master/defaults/exclude.txt as of 25/08/2020). This left 54 793 assemblies for downstream analysis. A full metadata table, list of acknowledgements and exclusions is provided in Table S3. The 54 793 SARS-CoV-2 assemblies were profile aligned against Wuhan-Hu-1 (GenBank accession no. MN908947.3) using mafft v.7.205 [29].

### Phylogenetic reconstruction

**Fig. 2.**
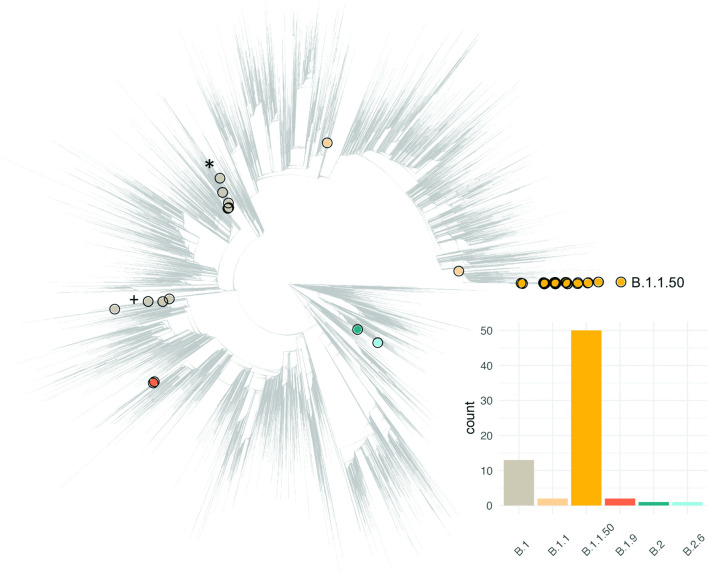
Phylogenetic placement of genomes generated in this study highlighted in the context of a large global phylogeny of 54 804 SARS-CoV-2 sampled up to August 2020. The phylogeny is depicted in grey, with tips highlighted for SARS-CoV-2 genomes sampled in Palestine. The colour provides the pango lineage assignment, the frequency and colour of which are denoted in the bar plot at bottom right. An equivalent phylogeny highlighting continental regions is provided in Fig. S3. + and * denote specific lineages discussed in the text.

The 69 aligned high-coverage Palestinian sequences generated herein and 54 793 strains from the worldwide diversity were concatenated and a maximum-likelihood tree built using iq-tree 2.1.0 Covid release [30]. A further 57 long-branch phylogenetic outliers were removed following application of TreeShrink [31] (given in Table S3). The final tree of 54 804 samples, rooted on Wuhan-Hu-1, is provided in Figs 2 and S3. Trees were queried and plotted using the R packages Ape v5.4 [32] and ggtree v1.16.6 [33] (Figs S3–S8).

### Lineage assignment and mutation analysis

pango lineages were assigned to each of the Palestinian SARS-CoV-2 assemblies using the dynamic nomenclature tool Pangolin [34] (https://github.com/cov-lineages/pangolin, applied 28/8/2020). The nucleotide positions of SNPs identified in the 69 assemblies are provided in Table S2, with annotations relative to Wuhan-Hu-1. The number of SNPs differing between viral assemblies within Palestine and across global datasets was assessed using SNP-sites [35] and SNP-dists (https://github.com/tseemann/snp-dists), with heatmaps plotted using ComplexHeatMap v2.1.2 [36] (Figs S2 and S9).

### Phylogenetic dating

To estimate the age of the largest transmission cluster (Figs S7–S10), we extracted a subset of 1252 B.1.1 SARS-CoV-2 from the phylogeny including the B.1.1.50 clade. The BactDating [37] *roottotip*() function was applied to compute the root-to-tip temporal regression for both the global tree and subsets of trees (Figs S11 and S12). In all cases, significance was assessed following 10 000 random permutations of sampling dates. Confidence intervals (CIs) around the inferred rates were assessed through 1000 bootstrap resamples with replacement. As with the global tree, the subset B.1.1 clade exhibited measurable evolution through time both with and without the earliest SARS-CoV-2 genome (reference Wuhan-Hu-1) included (*P*<1×10^−4^ in all cases). Following confirmation of significant temporal signal, we applied *dater*() within the TreeDater package v0.50 specifying a strict clock model and assessed CIs following 100 iterations of the *parboot*() parametric bootstrap fitting method (Fig. S13). Tip-dated phylogenetic trees, together with associated CIs, were assessed and plotted using ggtree v1.16.6 [33].

## Results

### Palestine SARS-CoV-2 samples fall across the diversity of global clusters

Our data comprise 69 SARS-CoV-2 genome samples spanning from the early stages of the COVID-19 epidemic in Palestine from March 2020 through until late August 2020, collected in 17 locations (seven governorates) (Fig. 1a–e, Table S1). The mean difference between any two samples was 11.6 mutations (95% CI 8.13–18.10), though with detectable structure in SNP sharing patterns often following the governorate of sampling (Fig. S2). A total of 67/69 assemblies carried the spike protein mutation D614G, with 65 carrying the full four mutation D614G haplotype (nucleotide positions 241, 3037, 14 408, 23 403). A total of 52 also carried the three neighbouring mutations in the nucleocapsid protein (28 881–28 883) and 1 sample (35) carried an 11 nucleotide insertion (frameshift) at position 27 301 (Orf6). A complete list of mutations carried by each genome, including synonymous and nonsynonymous status, is provided in Table S2.

When placed in a large global phylogeny of SARS-CoV-2, the 69 sequences from Palestine fall into six pango lineages, defined by the Pangolin dynamic lineage classification tool [34], interspersed over the global phylogenetic tree (Figs 1 and 2, Table S1). This includes five ‘singletons’ that are phylogenetically unrelated to any other SARS-CoV-2 genome obtained from Palestine, as well as a pair of related samples assigned to the B.1.9 lineage. The pairwise SNP differences across a random sub-sample of the global alignment (mean 12.8; 95 % CI 8.2–19.4) show no significant differences to those observed in Palestine, meaning our dataset can be considered as a representative random sample of the SARS-CoV-2 genomic diversity in circulation globally.

### From the global to the local

We additionally observe three phylogenetic clades comprising multiple (≥6) closely related SARS-CoV-2 strains sampled in Palestine (Fig. 2). This includes two distinct B.1-associated lineages of six strains and one large cluster of B.1.1.50 SARS-CoV-2 (Fig. 2). The first B.1-associated clade (flagged with an asterisk in Fig. 2) comprises five samples from Bethlehem and one from Ramallah spanning from the 4th of March 2020 through to the 29th of March 2020. Three samples are zero SNPs apart (sample identifiers: 28, 16, 19) with two collected in Bethlehem, both on the 16th of March 2020, and one from Ramallah 13 days later (Fig. S4). These three samples fall within a cluster of 110 SARS-CoV-2 that are genetically strictly identical despite having been sampled in 21 different countries between the 4th of March to 26th of April 2020 (Fig. S5). Within this cluster, we identify that samples 16 and 28 share 29 minority variants (among which 2 are found only in those two strains), which may be indicative of local transmission. The second B.1-associated clade (flagged with a plus sign in Fig. 2) of six samples includes two SARS-CoV-2 sampled from Tulkarem on the 4th of March (genetically identical), two samples from Bethlehem and two from Jerusalem all sampled on the 31st of March 2020 (Fig. S6).

### B.1.1.50 transmission cluster

The majority of our Palestinian samples (*n*=50; 73%), however, fall into a single, tight clade of closely related strains also including one sample from the UK and eight from Israel (Figs 2, S3 and S7) spanning a collection period from March 2020 to August 2020 (Fig. S8), and encompassing a mean of 8.5 (95% CI 5.8–14.6) pairwise SNPs (Fig. S9). Our data falling within this lineage include 57 unique nonsynonymous mutations, with all members of the lineage harbouring nonsynonymous changes at nucleotide positions 14 408, 15 438, 23 403, 25 785, 28 881 and 28 883 (Table S2, Fig. S10). While we do not detect significant accumulation of mutations over the sampling period in this clade, both the global phylogeny and a subset sample of 1220 B.1.1 genomes including this clade exhibit a highly significant temporal signal following randomizations of sampling date (*P*<1×10^−4^) (Figs S11 and S12). We estimate the rate, through linear regression, over the global alignment to be 25.1 (23.3–27.2) substitutions per genome per year, with significantly slower estimates of 18.3 (17.3–18.9) for the sub-sampled B.1.1 group, suggesting some slowing of rates through time since the beginning of the pandemic [38, 39].

**Fig. 3.**
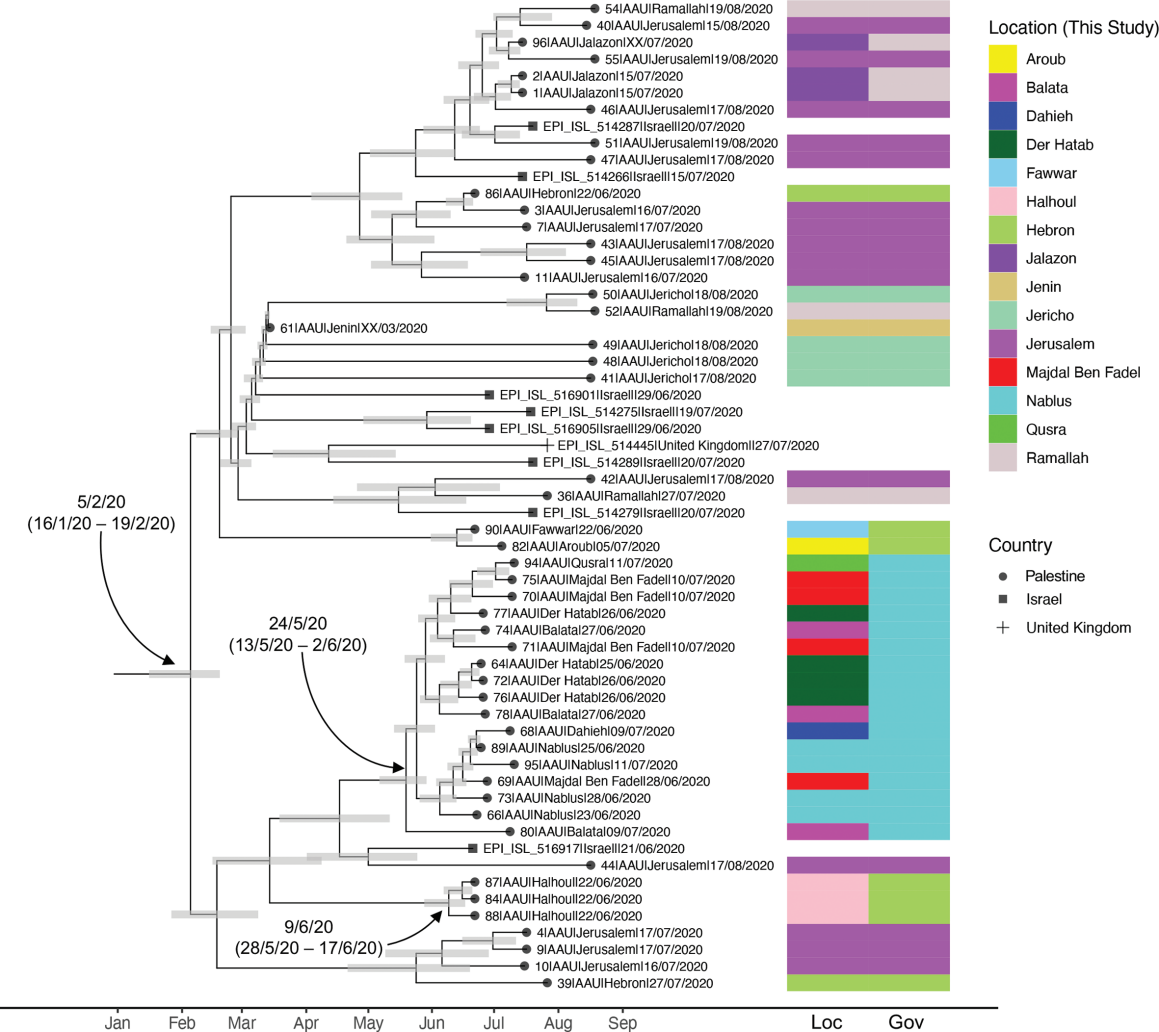
Time calibrated phylogenetic tree for the closely related B.1.1.50 clade subset from 1252 B.1.1 genomes. The coloured panel on the right of the tree provides the location (Loc) and governorate (Gov) of samples generated in this study (Palestine). Samples without colour panels derive from Israel and the UK, as shown in the tip labels. Grey bars provide the 95% CIs around the estimated age of phylogenetic nodes.

To formally estimate the age of the common ancestor of the B.1.1.50 clade, dominated by samples from our study, we applied TreeDater [40] to the subsampled B.1.1 phylogeny (Fig. S13). This allowed us to estimate the age of the node giving rise to the B.1.1.50 grouping to the 5th of February 2020 (16th January–19th February 2020; CIs following parametric bootstrapping) (Fig. 3). This suggests that Palestinian strains belonging to this clade were being transmitted locally already around these dates. Our timed phylogeny further allows estimation of the lower bound of the date of introduction into various local regions. For example, the collection of samples from the Nablus governorate shares an estimated most recent common ancestor dating to the 24th of May 2020 (13th of May–2nd of June 2020), and the three genomes sampled from patients on the 22nd of June 2020 in Halhoul share an estimated ancestor dating to the 9th of June 2020 (28th May–17th June 2020).

### Intra-genome minor allelic diversity

After discarding minor alleles with an intra-individual frequency <0.05, which for most are likely to be spurious, 598 polymorphic sites were retained. The vast majority of minor alleles (96%) displayed frequencies 0.05<*x*<0.2 (Figs S14 and S15) and most of them were present only in one sample (Fig. S16). The low frequency of minor alleles shared between samples prevented us from using this signal to reconstruct transmission chains. Indeed, we found no statistically significant correlation between the SNP-based phylogenetic signal and that of minor alleles (*R* ^2^=7.7×10^−4^; permutation *P* value=0.94). The lack of congruence between SNP-based phylogenetic signal and the distribution of minor allele frequency variants suggests that it would be difficult to leverage the latter for the reconstruction of transmission chains in this case (Figs S17 and S18).

## Discussion

Phylogenetic analyses of SARS-CoV-2 genomes sampled in Palestine over the first epidemic wave point to an earlier introduction and circulation of the virus than had been previously recognized, in line with the situation in many other regions of the world. This suggests SARS-CoV-2 was in sustained circulation in the region prior to the establishment of public-health interventions. The local COVID-19 epidemic(s) in Palestine were seeded by multiple (at least nine) independent introductions of SARS-CoV-2, though the lack of geographical structure and incomplete sampling make it challenging to pinpoint the exact geographical sources of import events. The diversity of SARS-CoV-2 in circulation in Palestine during the first epidemic wave (Fig. 2) recapitulates at least some of the global diversity in the SARS-CoV-2 population, though we do identify instances of local community transmission, including the B.1.1.50 pango lineage.

One of the major challenges in reconstructing the spread, and formal direct transmissions, of SARS-CoV-2 is the relatively low mutation rate [1], meaning multiple transmissions can occur before any mutation is observed in the genome [41]. Our estimated rate over the global alignment of 25.1 (23.3–27.2) mutations per genome per year falls in line with other published rates, and remains consistent with mutation rates observed in other coronaviruses that are maintained relatively low due to the action of a proof-reading protein (non-structural polyprotein 14) [42]. Epidemiological reconstructions are further challenged by the rapid global dissemination of SARS-CoV-2 and marked imbalance in the genomic data available from different geographical regions. Therefore, care must be taken when assigning the geographical sources of cases [17, 41]. As an example, our dataset includes three samples falling into a B.1 clade of 110 genetically identical sequences sampled over 21 nations over the course of 53 days (Fig. S5).

A possible approach to reconstructing transmission in these settings has been suggested by the use of shared minority variants [18, 43, 44]. In our dataset, we do identify a set of three identical genomes, two of which share minority variants, suggesting these two samples are more closely related. However, overall, despite considerably deep sequencing of the samples in our dataset, we find no usable phylogenetic signal in minor allelic variants that may be leveraged to aid in the reconstruction of transmission chains. Indeed, we found no evidence for any correlation between pairwise genetic distance between samples and their propensity to share minority variants (Figs S16 and S17).

The majority of SARS-CoV-2 genomes in our Palestinian dataset fell into a single cluster of B.1.1.50 SARS-CoV-2. Not precluding the possibility of many unsampled cases, our phylogenetic analyses point to this phylogenetic grouping representing a major local transmission cluster that has accumulated diversity primarily within Palestine over the early epidemic wave of Spring 2020. At the time of analysis, this cluster included eight SARS-CoV-2 samples from Israel and one from a patient in the UK; with 288 and 18 139 representatives from these countries in our global dataset, respectively (current to 25 August 2020).

Using phylogenetic tip-dating approaches, we estimate the age of the B.1.1.50 lineage to early February 2020, predating the first reports of COVID-19 positive patients in Palestine in a hotel in Bethlehem, where a group of Greek tourists had visited the hotel in late February 2020 and were diagnosed with the virus, as well as confirmed cases of college students returning from Europe (Fig. 1a). This lineage was circulating in Palestine until at least the latter half of August of 2020. Consequently, other local clusters have emerged from within the initial B.1.1.50 clade, for example, the sub-lineage of B.1.1.50 circulating in the governorate of Nablus since mid to late May 2020 (Fig. 3). Of note, more recent assessments of the prevalence of differing SARS-CoV-2 lineages (February 2021) indicates B.1.1.50 has persisted, now comprising 556 genomes sampled in eight countries (https://cov-lineages.org/lineages/lineage_B.1.1.50.html, accessed 17/02/2021). This includes 423 genomes sampled in Israel and 54 from the UK.

Due to the limited geographical structuring of the global genetic distribution of SARS-CoV-2, it is difficult to confidently identify putative sources of introduction of the virus when sampling locally transmitting lineages. Thus, we can only speculate on the origin of B.1.1.50. On the basis of human movement, Israel and Europe provide the most plausible sources. Despite attempts by the Palestinian government to discourage its residents from crossing from and into Israeli areas, daily commuting of workers and residents between the West Bank and Israel never entirely ceased. Close to 2000 Palestinians entered the West Bank from Jordan via the Allenby crossing between the 1st and 13th of July. Another plausible source are Palestinian students returning from Europe, as well as the USA.

Our genomic analyses also pinpoint the presence of transmission lineages for which there are no known epidemiological links, for example, we identify a clear case of localized community transmission predating by at least weeks the earliest cases in Palestine. As such, our study supports the adoption of genomic surveillance in Palestine, highlighting the potential of genomic epidemiology to uncover and ultimately monitor patterns of disease spread at both global and local scales.

## Supplementary Data

Supplementary material 1

Supplementary material 2

Supplementary material 3
