# Supplementary material for: Genomic epidemiology of the first epidemic wave of severe acute respiratory syndrome coronavirus 2 (SARS-CoV-2) in Palestine
Source: Microb Genom. 2021 Jun 22;7(6):000584. doi: 10.1099/mgen.0.000584 (PMC8461465; doi:10.1099/mgen.0.000584)
Supplement: Supplementary material 1 [file mgen-7-0584-s001.pdf]

Supplementary Materials

Genomic epidemiology of the first epidemic wave of SARS-CoV-2 in Palestine

Table S1: SARS-CoV-2 genomes generated in this study. Metadata provides information on the date of collection, governorate and location. Pangolin lineage assignments are provided (29/8/2020) version with all assignments made with a probability score of 1. The percentage cover and mean depth compared to the reference genome Wuhan-Hu-1 is also provided. All genomes were generated at mean depth >20.

ID	Date	Region	Country	Governorate	Location	Pangolin_lineage	Percent_cover	Mean_depth	GISAID_ID
**1**	15/07/2020	Asia	Palestine	Ramallah	Jalazon	B.1.1.50	99.88	2092	EPI_ISL_596507
**2**	15/07/2020	Asia	Palestine	Ramallah	Jalazon	B.1.1.50	99.89	3824	EPI_ISL_596514
**3**	16/07/2020	Asia	Palestine	Jerusalem	Jerusalem	B.1.1.50	99.65	32	EPI_ISL_596522
**4**	17/07/2020	Asia	Palestine	Jerusalem	Jerusalem	B.1.1.50	99.85	164	EPI_ISL_596533
**7**	17/07/2020	Asia	Palestine	Jerusalem	Jerusalem	B.1.1.50	99.84	171	EPI_ISL_596555
**9**	17/07/2020	Asia	Palestine	Jerusalem	Jerusalem	B.1.1.50	99.71	76	EPI_ISL_596567
**10**	16/07/2020	Asia	Palestine	Jerusalem	Jerusalem	B.1.1.50	99.82	388	EPI_ISL_596500
**11**	16/07/2020	Asia	Palestine	Jerusalem	Jerusalem	B.1.1.50	99.97	7445	EPI_ISL_596501
**13**	04/03/2020	Asia	Palestine	Bethlehem	Bethlehem	B.1	99.9	3132	EPI_ISL_596502
**14**	10/03/2020	Asia	Palestine	Bethlehem	Bethlehem	B.1	99.94	8160	EPI_ISL_596503
**15**	07/03/2020	Asia	Palestine	Bethlehem	Bethlehem	B.1	99.26	1882	EPI_ISL_596504
**16**	16/03/2020	Asia	Palestine	Bethlehem	Bethlehem	B.1	99.67	47	EPI_ISL_596505
**19**	16/03/2020	Asia	Palestine	Bethlehem	Bethlehem	B.1	99.84	137	EPI_ISL_596506
**21**	19/03/2020	Asia	Palestine	Ramallah	Ramallah	B.1.1	99.85	806	EPI_ISL_596508
**22**	21/03/2020	Asia	Palestine	Bethlehem	Bethlehem	B.2.6	99.85	229	EPI_ISL_596509
**23**	22/03/2020	Asia	Palestine	Ramallah	Ramallah	B.1.9	99.79	182	EPI_ISL_596510
**24**	22/03/2020	Asia	Palestine	Ramallah	Ramallah	B.1.9	99.86	1090	EPI_ISL_596511
**28**	29/03/2020	Asia	Palestine	Ramallah	Ramallah	B.1	99.81	133	EPI_ISL_596512
**29**	31/03/2020	Asia	Palestine	Bethlehem	Bethlehem	B.1	99.85	288	EPI_ISL_596513
**30**	31/03/2020	Asia	Palestine	Bethlehem	Bethlehem	B.1	99.89	3041	EPI_ISL_596515
**31**	31/03/2020	Asia	Palestine	Jerusalem	Jerusalem	B.1	99.85	277	EPI_ISL_596516
**32**	31/03/2020	Asia	Palestine	Ramallah	Ramallah	B.2	99.94	4384	EPI_ISL_596517
**33**	31/03/2020	Asia	Palestine	Jerusalem	Jerusalem	B.1	99.93	3961	EPI_ISL_596518
**34**	04/03/2020	Asia	Palestine	Tulkarem	Tulkarem	B.1	99.63	40	EPI_ISL_596519
**35**	04/03/2020	Asia	Palestine	Tulkarem	Tulkarem	B.1	99.88	3086	EPI_ISL_596568
**36**	27/07/2020	Asia	Palestine	Ramallah	Ramallah	B.1.1.50	99.84	477	EPI_ISL_596520
**39**	27/07/2020	Asia	Palestine	Hebron	Hebron	B.1.1.50	99.85	625	EPI_ISL_596521
**40**	15/08/2020	Asia	Palestine	Jerusalem	Jerusalem	B.1.1.50	99.9	3074	EPI_ISL_596523
**41**	17/08/2020	Asia	Palestine	Jericho	Jericho	B.1.1.50	99.45	22	EPI_ISL_596524
**42**	17/08/2020	Asia	Palestine	Jerusalem	Jerusalem	B.1.1.50	99.79	78	EPI_ISL_596525
**43**	17/08/2020	Asia	Palestine	Jerusalem	Jerusalem	B.1.1.50	99.94	5867	EPI_ISL_596526
**44**	17/08/2020	Asia	Palestine	Jerusalem	Jerusalem	B.1.1.50	99.88	2289	EPI_ISL_596527
**45**	17/08/2020	Asia	Palestine	Jerusalem	Jerusalem	B.1.1.50	99.85	267	EPI_ISL_596528

**46**	17/08/2020	Asia	Palestine	Jerusalem	Jerusalem	B.1.1.50	99.85	548	EPI_ISL_596529
**47**	17/08/2020	Asia	Palestine	Jerusalem	Jerusalem	B.1.1.50	99.98	5714	EPI_ISL_596530
**48**	18/08/2020	Asia	Palestine	Jericho	Jericho	B.1.1.50	99.89	1136	EPI_ISL_596531
**49**	18/08/2020	Asia	Palestine	Jericho	Jericho	B.1.1.50	99.99	9400	EPI_ISL_596532
**50**	18/08/2020	Asia	Palestine	Jericho	Jericho	B.1.1.50	99.94	4895	EPI_ISL_596534
**51**	19/08/2020	Asia	Palestine	Jerusalem	Jerusalem	B.1.1.50	99.85	380	EPI_ISL_596535
**52**	19/08/2020	Asia	Palestine	Ramallah	Ramallah	B.1.1.50	99.85	802	EPI_ISL_596536
**54**	19/08/2020	Asia	Palestine	Ramallah	Ramallah	B.1.1.50	99.86	1367	EPI_ISL_596537
**55**	19/08/2020	Asia	Palestine	Jerusalem	Jerusalem	B.1.1.50	99.84	738	EPI_ISL_596538
**60**	XX/03/2020	Asia	Palestine	Ramallah	Ramallah	B.1.1	99.88	1410	EPI_ISL_596539
**61**	XX/03/2020	Asia	Palestine	Jenin	Jenin	B.1.1.50	99.85	315	EPI_ISL_596540
**62**	XX/03/2020	Asia	Palestine	Jenin	Jenin	B.1	99.85	498	EPI_ISL_596541
**64**	25/06/2020	Asia	Palestine	Nablus	Der Hatab	B.1.1.50	99.84	244	EPI_ISL_596542
**66**	23/06/2020	Asia	Palestine	Nablus	Nablus	B.1.1.50	99.84	374	EPI_ISL_596543
**68**	09/07/2020	Asia	Palestine	Nablus	Dahieh	B.1.1.50	99.85	1763	EPI_ISL_596544
**69**	28/06/2020	Asia	Palestine	Nablus	Majdal Ben Fadel	B.1.1.50	99.85	837	EPI_ISL_596545
**70**	10/07/2020	Asia	Palestine	Nablus	Majdal Ben Fadel	B.1.1.50	98.06	22	EPI_ISL_596546
**71**	10/07/2020	Asia	Palestine	Nablus	Majdal Ben Fadel	B.1.1.50	99.9	5070	EPI_ISL_596547
**72**	26/06/2020	Asia	Palestine	Nablus	Der Hatab	B.1.1.50	99.86	3323	EPI_ISL_596548
**73**	28/06/2020	Asia	Palestine	Nablus	Nablus	B.1.1.50	99.86	1227	EPI_ISL_596549
**74**	27/06/2020	Asia	Palestine	Nablus	Balata	B.1.1.50	99.82	259	EPI_ISL_596550
**75**	10/07/2020	Asia	Palestine	Nablus	Majdal Ben Fadel	B.1.1.50	99.7	92	EPI_ISL_596551
**76**	26/06/2020	Asia	Palestine	Nablus	Der Hatab	B.1.1.50	99.84	364	EPI_ISL_596552
**77**	26/06/2020	Asia	Palestine	Nablus	Der Hatab	B.1.1.50	99.86	470	EPI_ISL_596553
**78**	27/06/2020	Asia	Palestine	Nablus	Balata	B.1.1.50	99.7	94	EPI_ISL_596554
**80**	09/07/2020	Asia	Palestine	Nablus	Balata	B.1.1.50	99.78	162	EPI_ISL_596556
**82**	05/07/2020	Asia	Palestine	Hebron	Aroub	B.1.1.50	99.89	5814	EPI_ISL_596557
**84**	22/06/2020	Asia	Palestine	Hebron	Halhoul	B.1.1.50	99.84	253	EPI_ISL_596558
**86**	22/06/2020	Asia	Palestine	Hebron	Hebron	B.1.1.50	99.74	146	EPI_ISL_596559
**87**	22/06/2020	Asia	Palestine	Hebron	Halhoul	B.1.1.50	99.85	641	EPI_ISL_596560
**88**	22/06/2020	Asia	Palestine	Hebron	Halhoul	B.1.1.50	99.7	56	EPI_ISL_596561
**89**	25/06/2020	Asia	Palestine	Nablus	Nablus	B.1.1.50	99.7	93	EPI_ISL_596562
**90**	22/06/2020	Asia	Palestine	Hebron	Fawwar	B.1.1.50	99.78	134	EPI_ISL_596563
**94**	11/07/2020	Asia	Palestine	Nablus	Qusra	B.1.1.50	99.81	309	EPI_ISL_596564
**95**	11/07/2020	Asia	Palestine	Nablus	Nablus	B.1.1.50	99.81	236	EPI_ISL_596565
**96**	XX/07/2020	Asia	Palestine	Ramallah	Jalazon	B.1.1.50	99.87	2100	EPI_ISL_596566

Table S2: [External file] List of SNPs identified in all the 69 isolates compared to the Wuhan-Hu-1 reference sequence (GenBank accession MN908947; equivalent GISAID ID EPI_ISL_402125). SNPs flagged as putative sequencing errors were discarded (a full list of 'masked' sites is available at https://github.com/W-L/ProblematicSites_SARS-CoV2/blob/master/problematic_sites_sarsCov2.vcf, accessed 25/08/2020).

Table S3: [External file] SARS-CoV-2 accessions included in global phylogenetic analysis. Full metadata is provided as downloaded directly from GISAID, with contributing, originating and submitting laboratories. Those accessions removed by TreeShrink as phylogenetic outliers are provided on a seperate tab

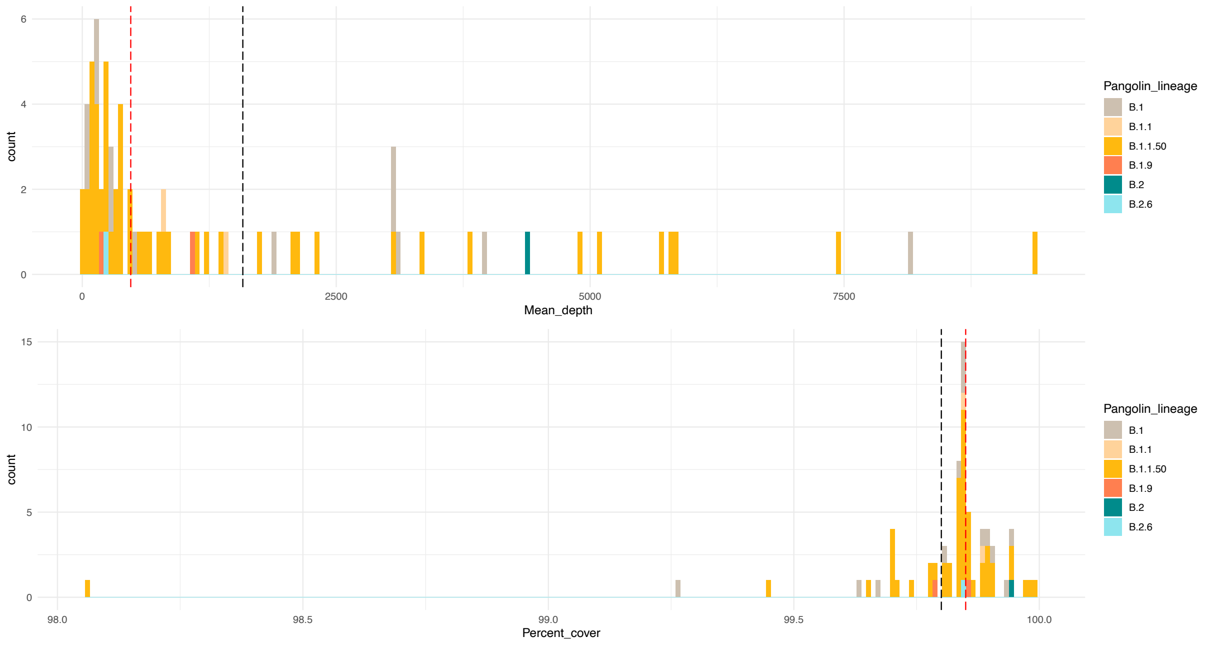

Figure S1 Histograms providing the mean depth of coverage (top) and percentage cover (bottom) of the Palestinian SARS-CoV-2 dataset. Raw values are provided in **Table S1**. The black dashed line provides the mean value and the red dashed line the median value in each case.

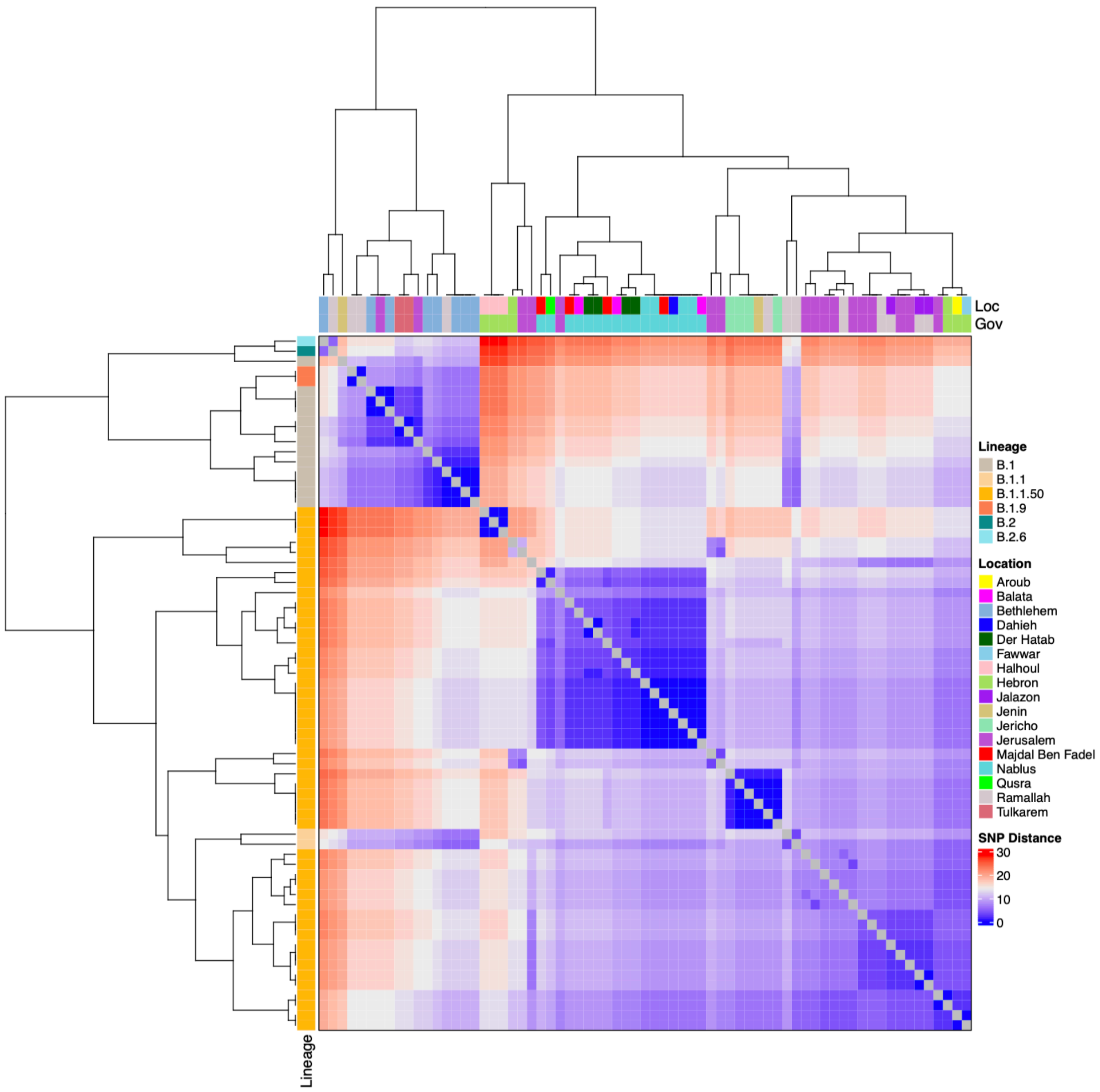

Figure S2 Pairwise SNP differences between all SARS-CoV-2 data collected in this study. Colour provides the number of SNPs differing as per the legend at right. The top panel provides the location 'Loc' and governorate 'Gov' where samples were collected and the left the PANGO lineage assigned.

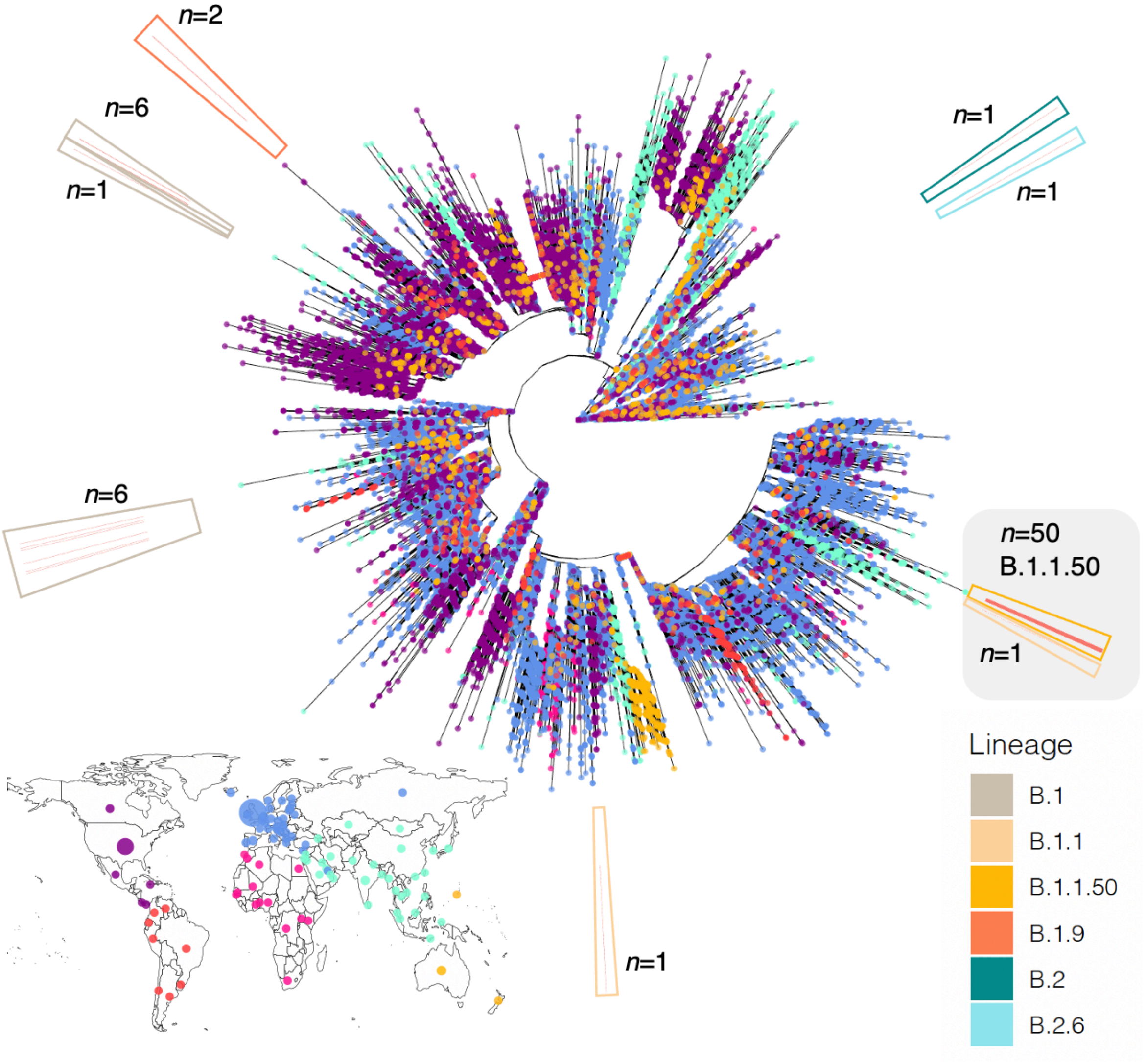

Figure S3 Phylogenetic placement of data collected in this study in the context of a large global phylogeny of 54,804 SARS-CoV-2 assemblies. Tip colour provides the continental region of sampling as given by the map at bottom left. The outer ring highlights the number of samples in different phylogenetic clades with the outer border providing the PANGO lineage assignments as per the key at bottom right. The B.1.1.50 lineage discussed in text is highlighted in grey.

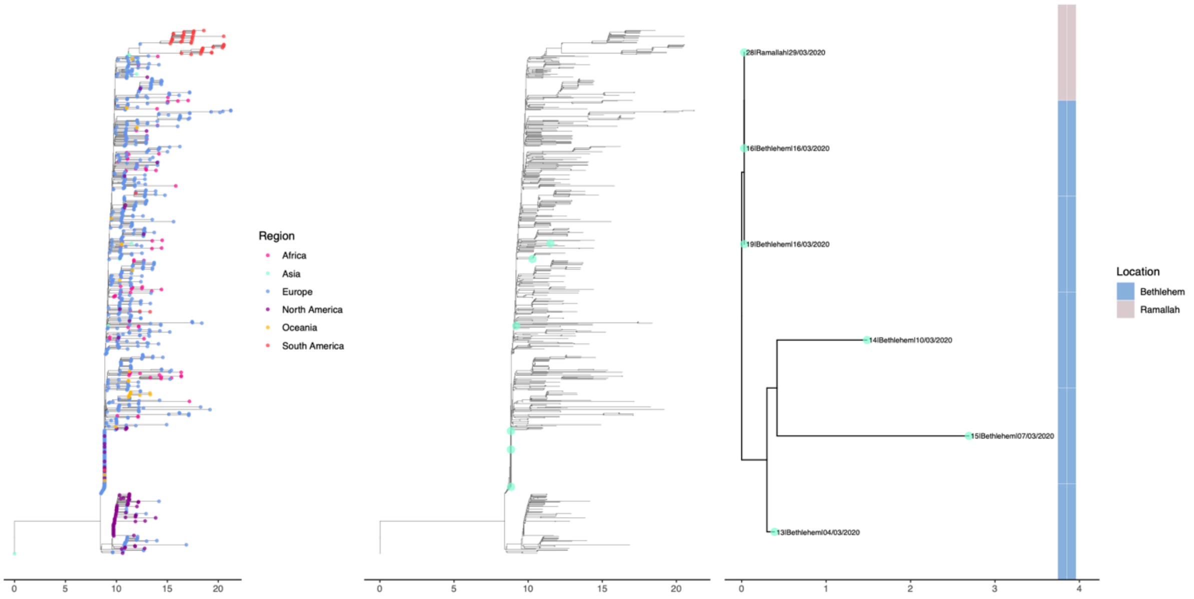

Figure S4 B.1 node extracted from the global SARS-CoV-2 phylogenetic tree including six samples from this study. The tree at left provides the global phylogenetic context with samples from this study highlighted in the middle panel. The panel at right provides a subset of the tree to demonstrate the diversity amongst our samples with the coloured bar providing the location/governorate.

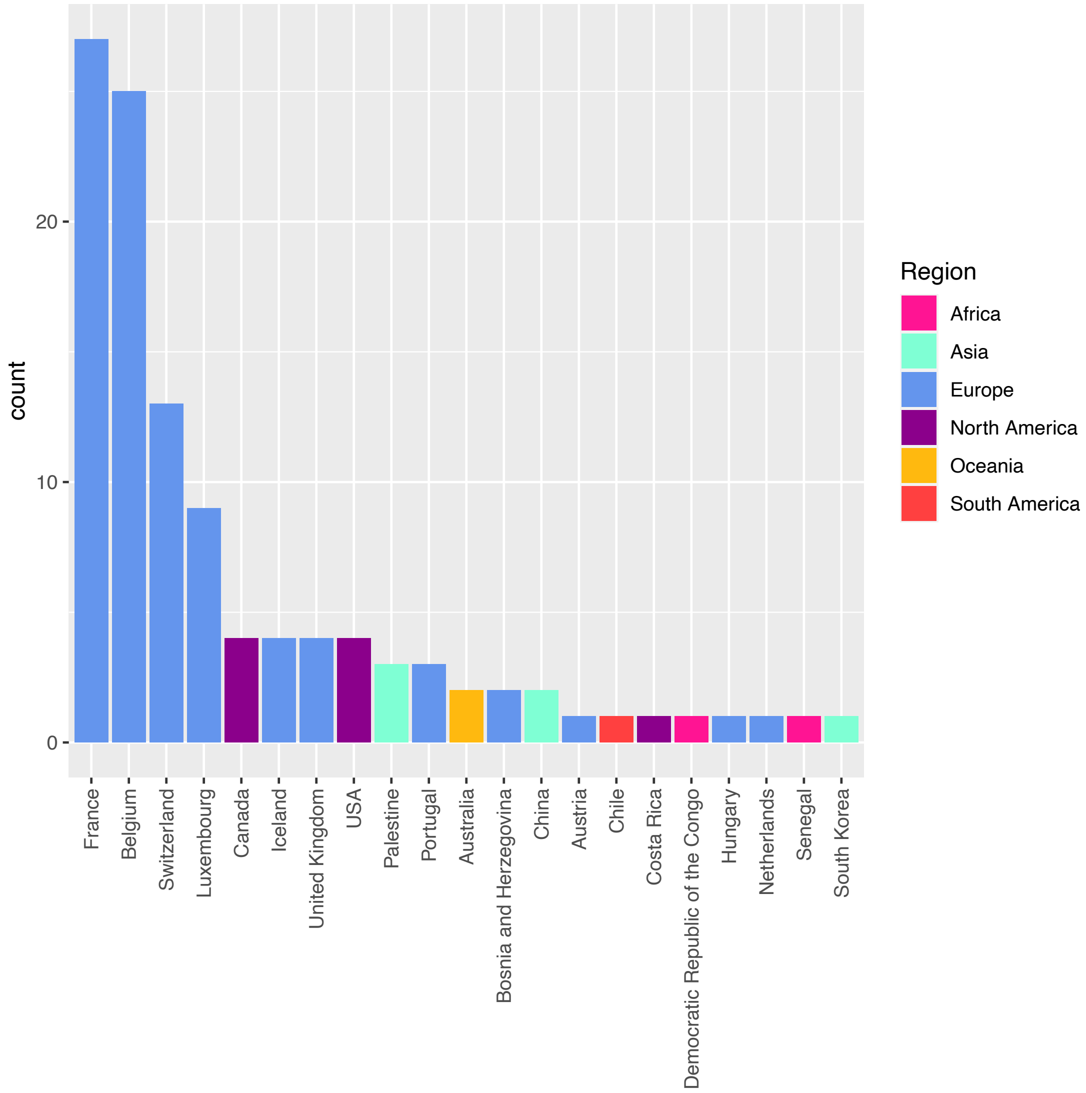

Figure S5 Geographic origin of the of the110 SARS-CoV-2 strains with identical genome sequences (B.1 lineage) including three Palestinian samples.

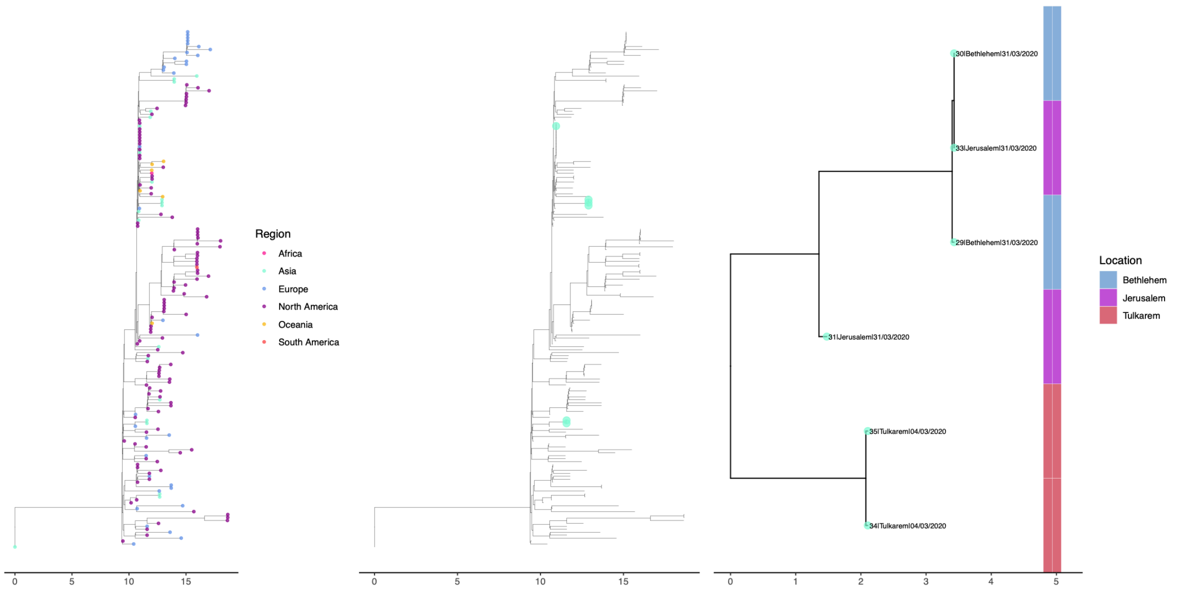

Figure S6 B.1 node extracted from the global SARS-CoV-2 phylogenetic tree including six samples from this study. The tree at left provides the global phylogenetic context with samples from this study highlighted in the middle panel. The panel at right provides a subset of the tree to demonstrate the diversity amongst our samples with the coloured bar providing the location/governorate.

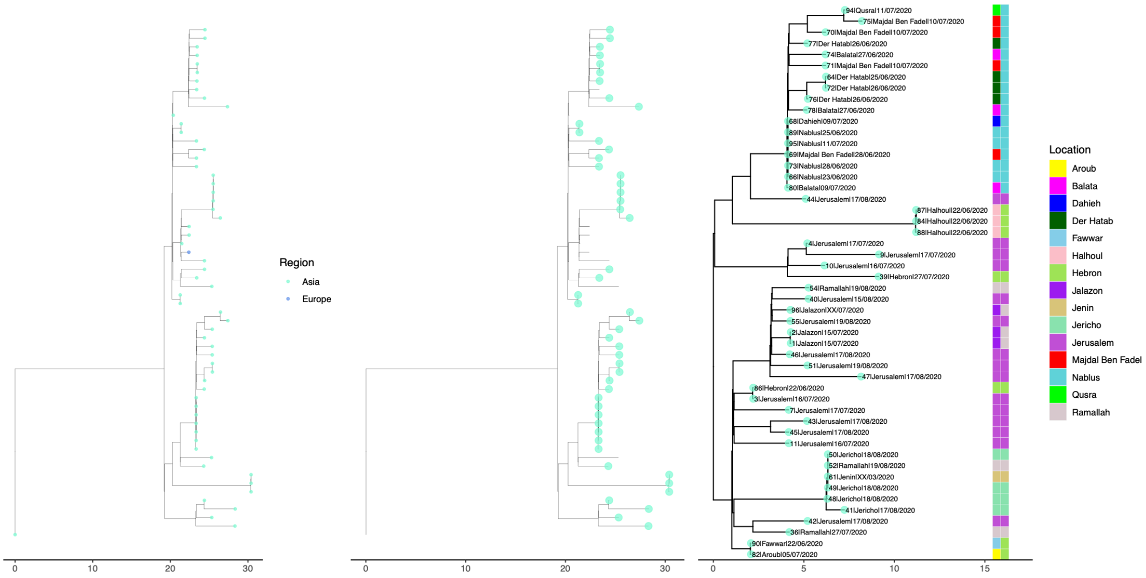

Figure S7 B.1.1.50 node extracted from the global SARS-CoV-2 phylogenetic tree including fifty samples from this study. The tree at left provides the global phylogenetic context with samples from this study highlighted in the middle panel. The panel at right provides a subset of the tree to demonstrate the diversity amongst our samples with the coloured bar providing the location/governorate.

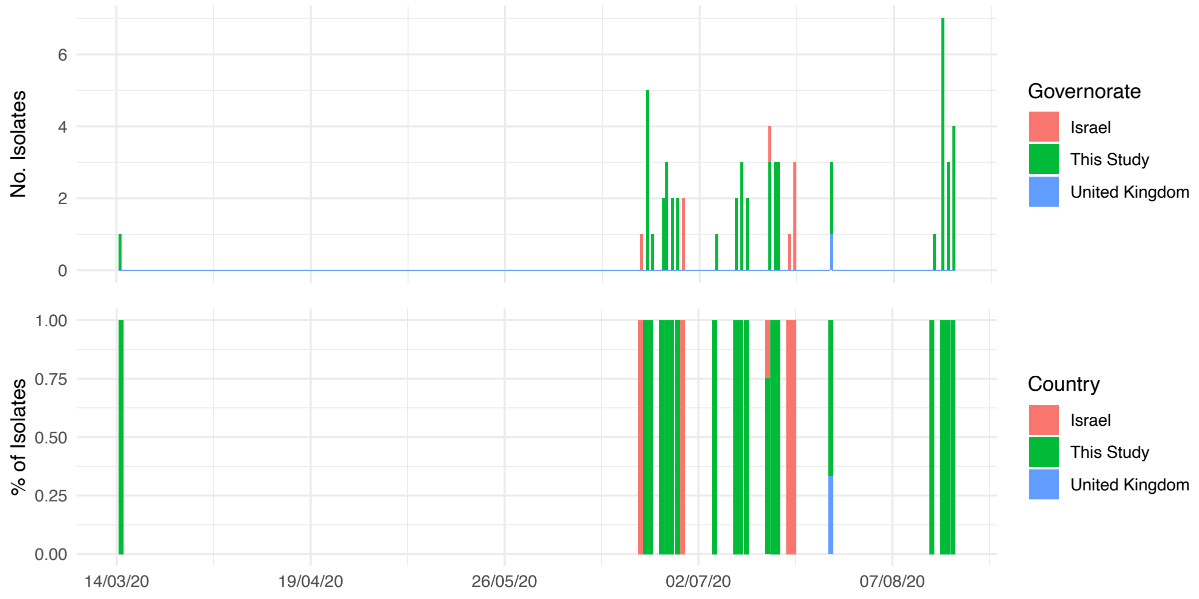

Figure S8 Sample collection dates and nation of isolation for SARS-CoV-2 isolates falling within the B.1.1.50 local phylogenetic cluster (**Figure S7**).

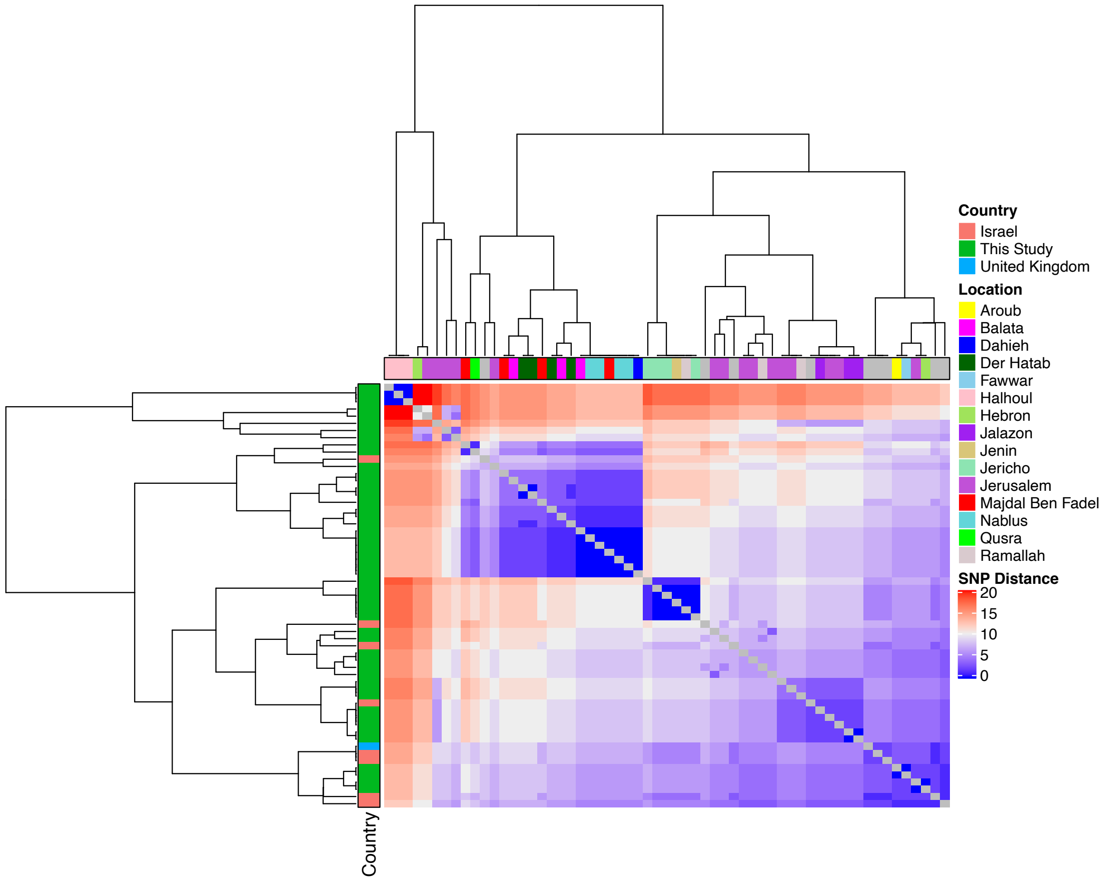

Figure S9 Pairwise SNP differences between SARS-CoV-2 falling within the B1.1.50 local cluster, including 50 SARS-CoV-2 genomes generated in this study. The colour scale provides the number of SNPs differing as per the legend at right. The top panel provides the location within Palestine (samples not generated in this study set to grey) where samples were collected. The panel at left provides the country assignment (nine global samples from Israel [n=8] and the UK [n=1]).

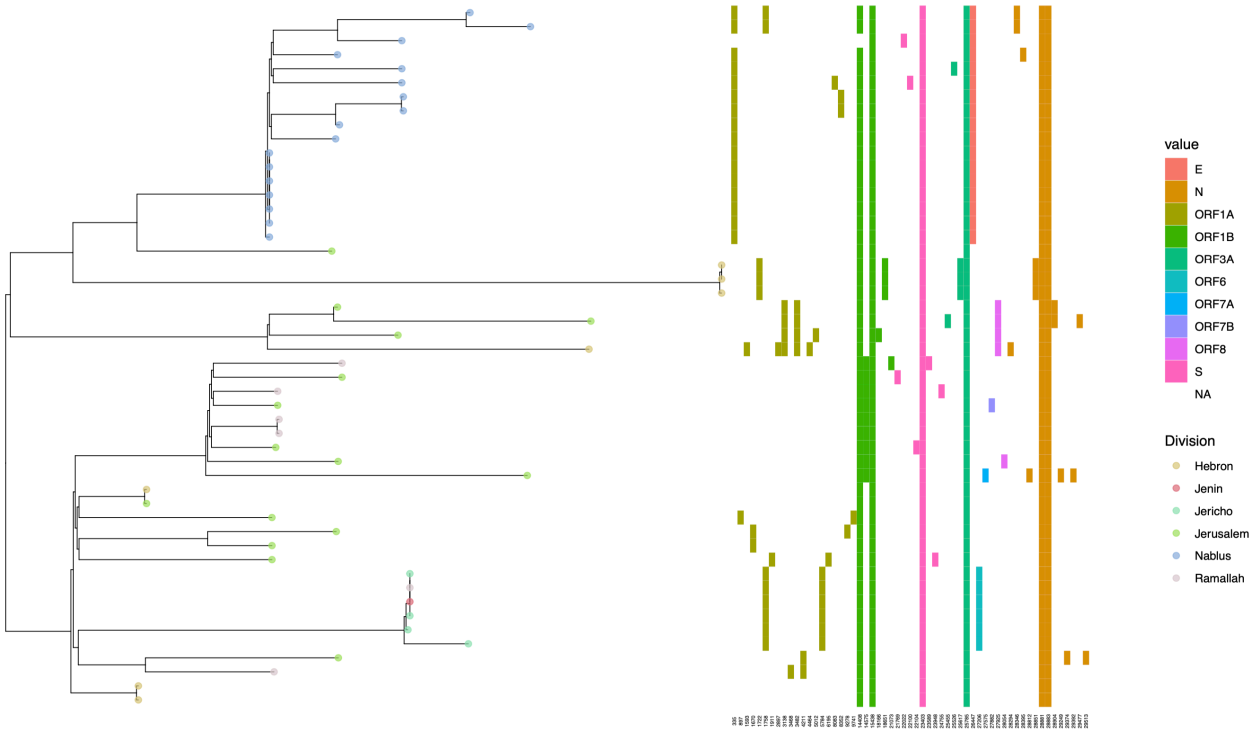

Figure S10 Distribution of nonsynonymous mutations observed in the B.1.1.50 Palestinian data, provided by colour (presence) heatmap ordered by the B.1.1.50 extracted maximum likelihood phylogeny. Full mutations are provided in **Table S2**.

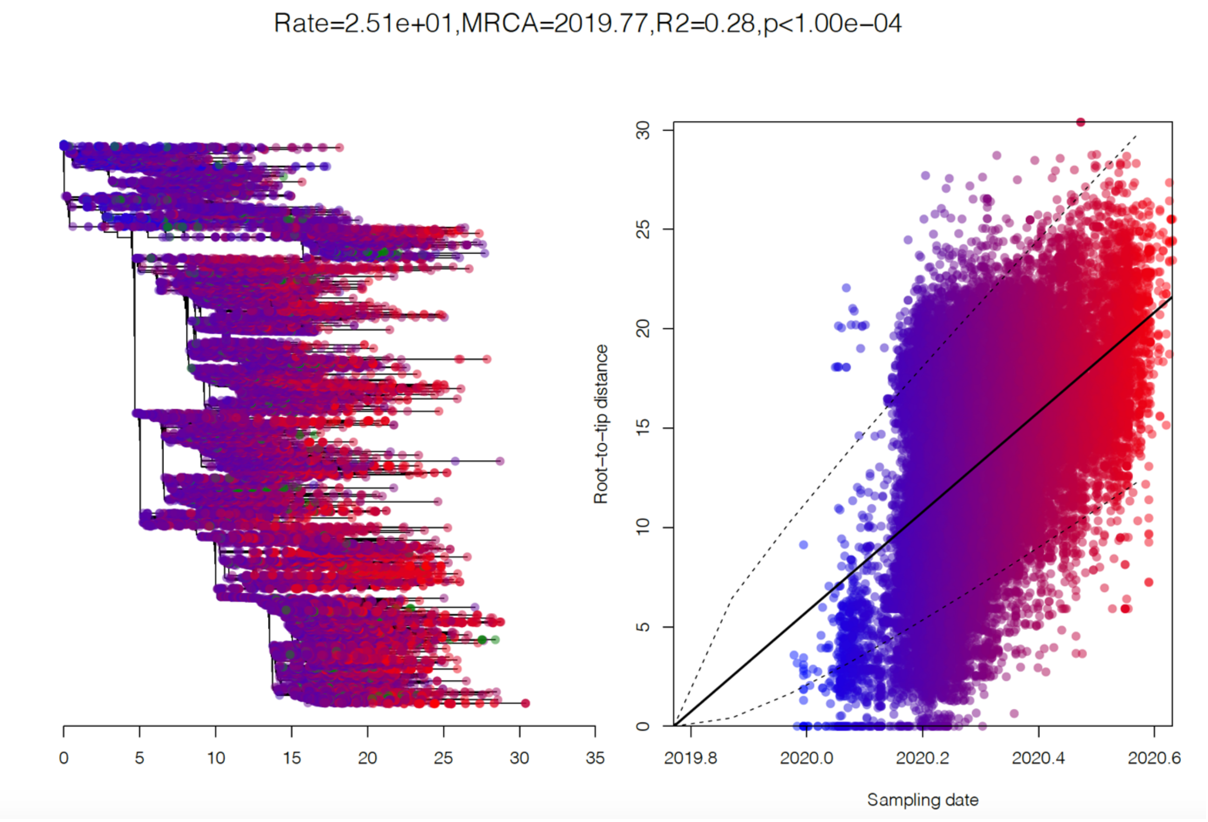

Figure S11 (left) Global phylogenetic tree rooted on Wuhan-Hu-1. (right) Correlation between the decimal date of sample collection (x-axis) and root-to-tip phylogenetic distance (y-axis) across the global dataset, with points coloured by location of collection. A significant temporal regression was obtained following 10,000 randomisations of collection date (MRCA 2019.77, r^2=0.276, p<1e-4). Estimated rates following 1000 bootstrap resamplings gave rise to values of 25.1 (23.3 – 27.2) subsitutions per genome per year.

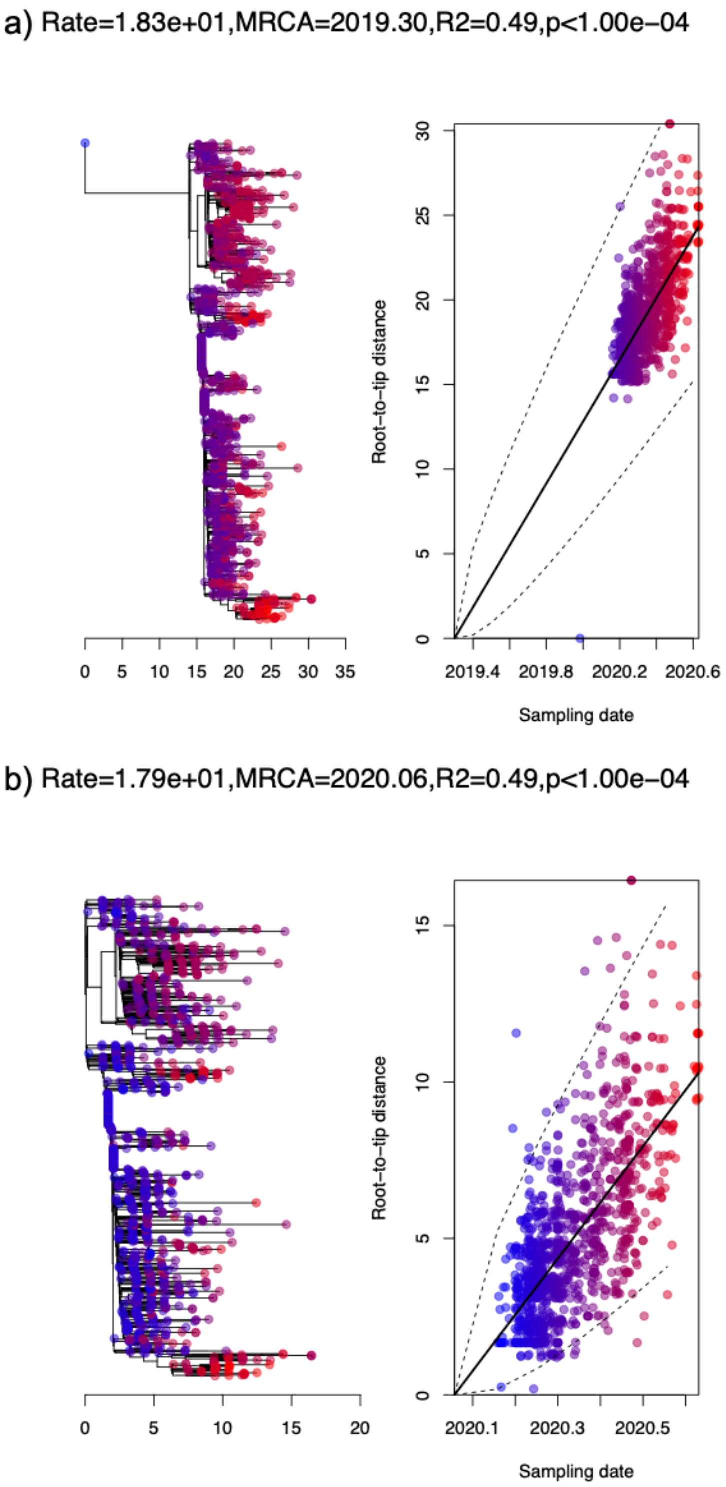

Figure S12: Subset phylogenetic tree of 1,252 B.1.1 genomes. a) Left panel provides the phylogenetic tree rooted on Wuhan-Hu-1. The right panel provides the root-to-tip phylogenetic distance (y) as a produce of the time of sampling (x), displaying a significant regression of rate 18.3 mutations per genome per year. b) Left panel provides the phylogenetic tree now dropping Wuhan-Hu-1. The right panel provides the root-to-tip phylogenetic distance (y) as a produce of the time of sampling (x), displaying a significant regression of rate 17.9 mutations per genome per year. In both cases *p*-values are provided following testing against 10,000 randomisations of the sampling date.

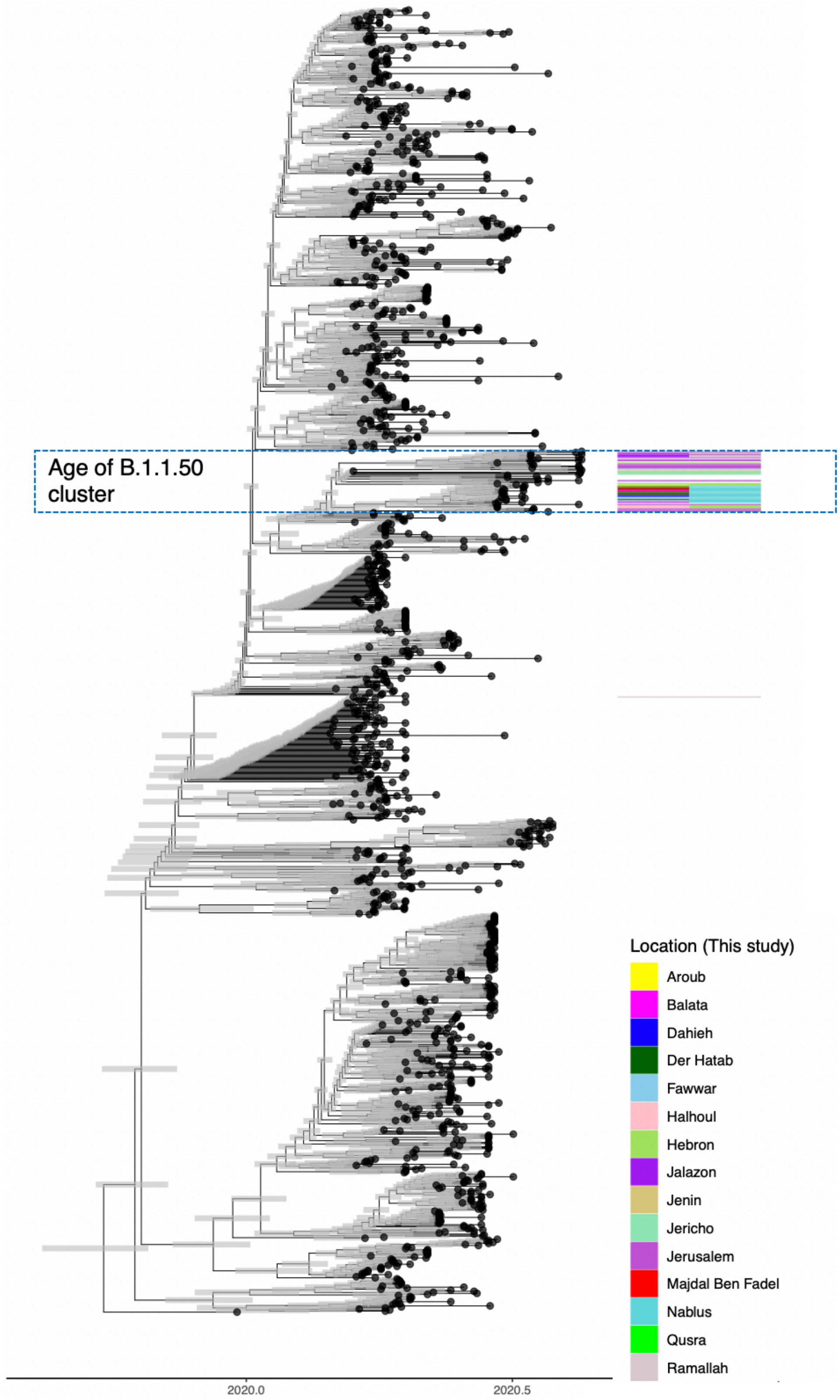

Figure S13: TreeDater time calibrated phylogenetic tree of 1252 B.1.1 SARS-CoV-2 genomes. Data from this study (Palestine) are highlighted by the coloured bars which give the location and governorate of patient location. Grey bars provide the 95% confidence intervals following parametric bootstrapping. The highlighted age of the B.1.1.50 clade dominated by data from our study is 5th February (16th January – 19th of February) (main text **Figure 3**).

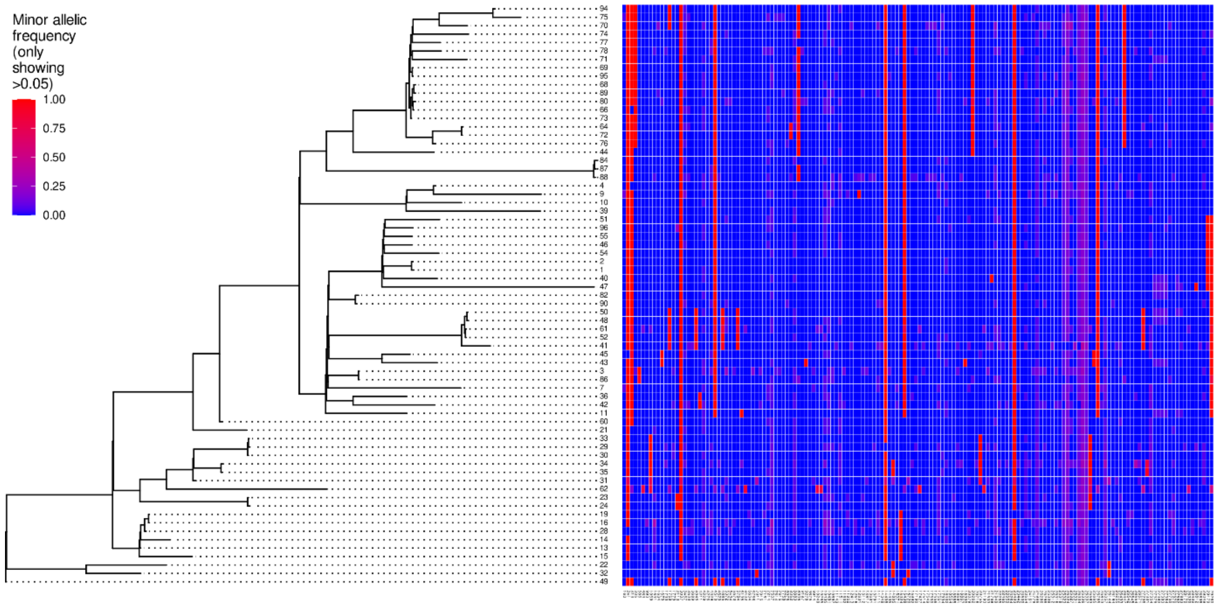

Figure S14: Alignment of the 69 genomes from Palestine restricted to the 167 sites with derived allele at frequency >0.05 in at least two isolates. The frequency of the minor variants is shown as a heat map. the vast majority of minor alleles (96%) displayed frequencies 0.05<x<0.2. Derived alleles near or at fixation are displayed in red.

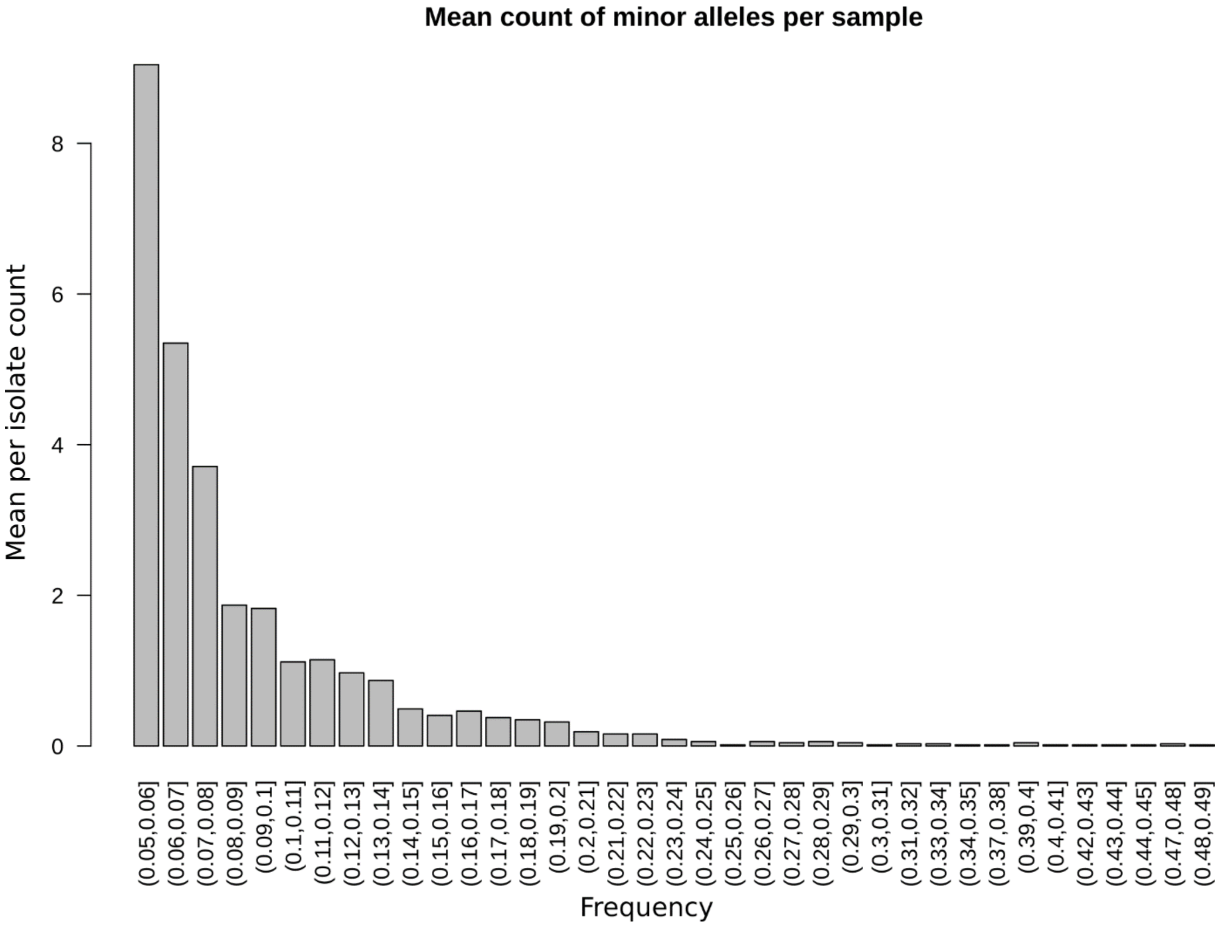

Figure S15: Distribution of the frequency of minor allele variants of within host polymorphisms found at a frequency above 0.05.

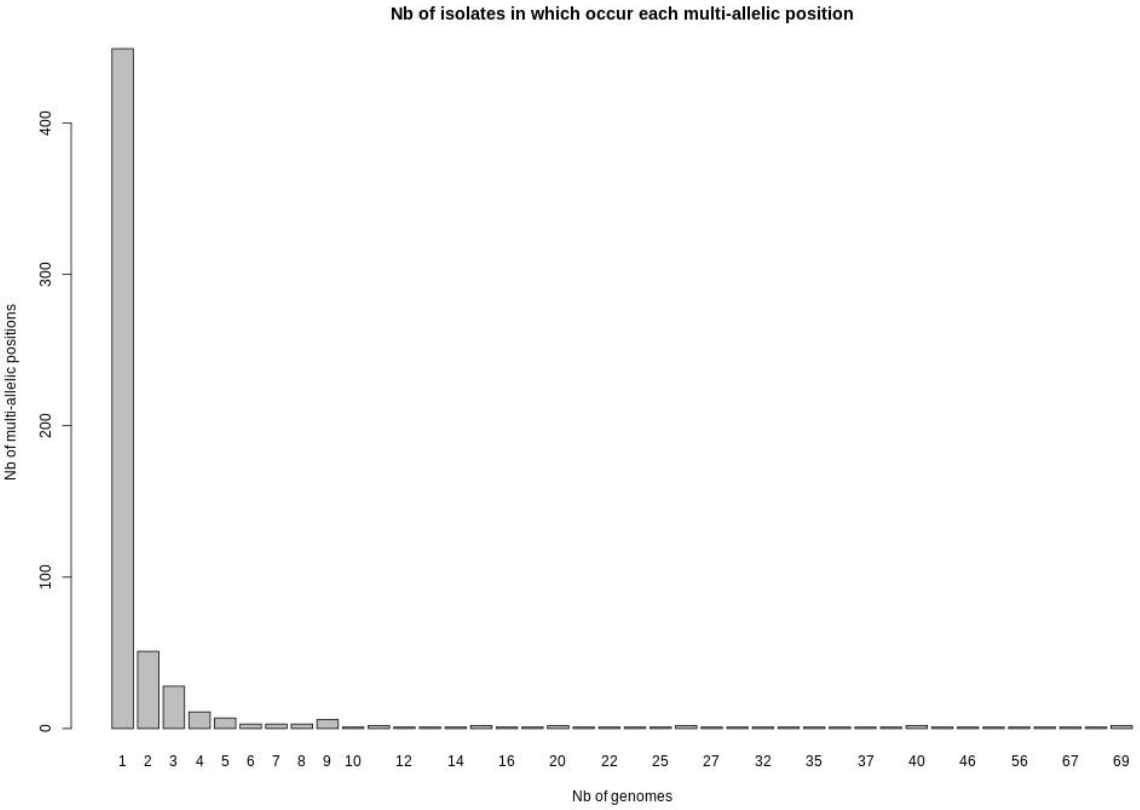

Figure S16: Distribution of the number of SARS-CoV-2 genomes sharing individual minor allele variants at a frequency above 0.05.

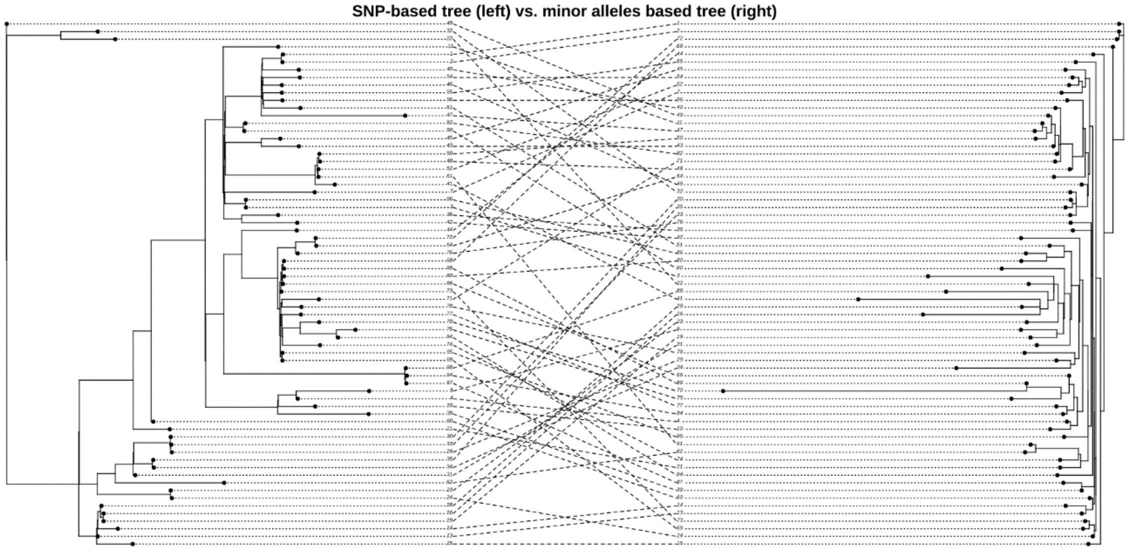

Figure S17: Cophyloplot showing the correspondence of the position of the strains in a maximum-likelihood SNP-based tree (left) and a maximum-likelihood minor allele-based tree (right). The minor allele-based tree is based on the binary matrix of presence or absence of a minor allele (frequency >0.05) at each nucleotide position of the genome.

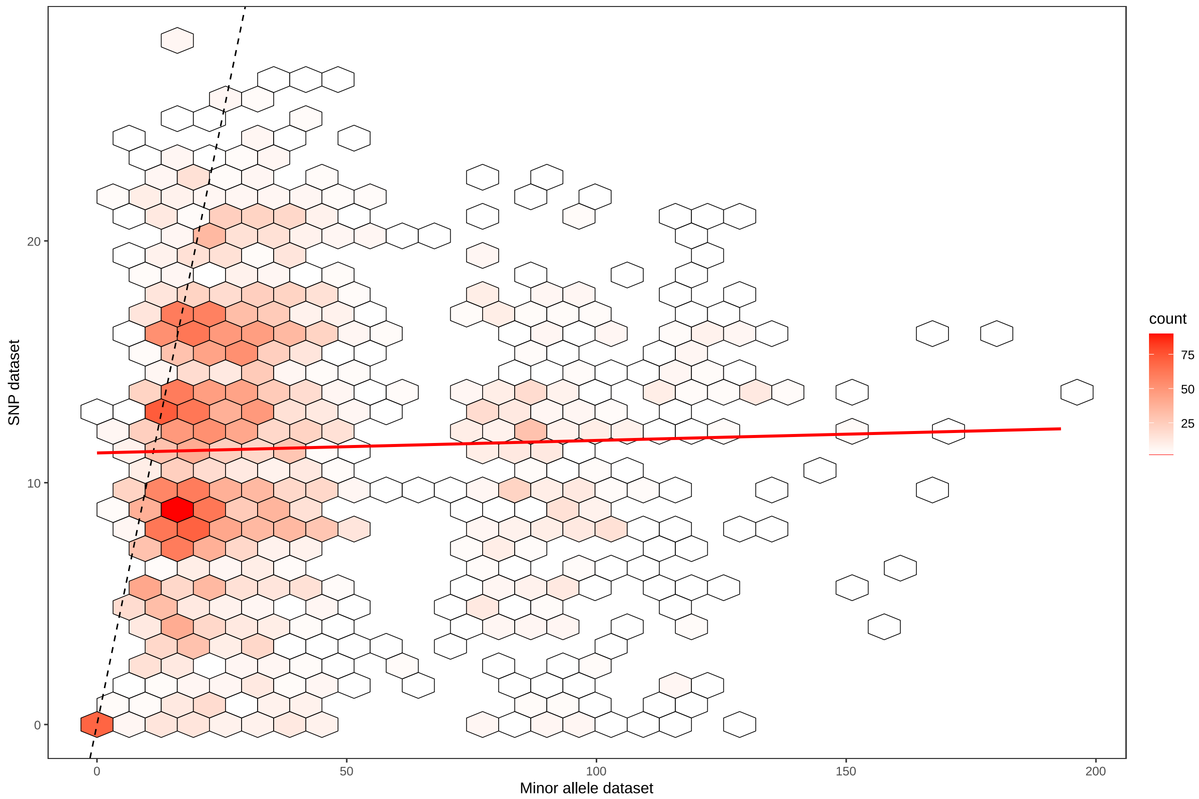

Figure S18; Correlation between the pairwise distances between SARS-CoV-2 genomes in the SNP-based alignment and the minor-allele based alignment matrices. Red line : linear regression ; dashed line : y=x. The relation between SNP-based and minor-allele paiwise genetic distances was not statistically signioficant ($R2 = 7.7e^{-4}$; Mantel test *p*-value=0.94).
